# Coronary sinus diameter to estimate congestion and predict survival

**DOI:** 10.1016/j.ijcha.2023.101294

**Published:** 2023-11-08

**Authors:** Agatella Barchitta, Giacomo Rossitto, Luisa Ruzza, Daniele Maio, Giuseppe Scaparotta, Domenico Bagordo, Francesco Antonini Canterin, Piergiuseppe Piovesana, Teresa Maria Seccia, Federico Nalesso, Lorenzo Calò, Gian Paolo Rossi

**Affiliations:** a University of Padova, Emergency Medicine and Hypertension, University Hospital, Padova, Italy; b School of Cardiovascular & Metabolic Health, University of Glasgow, Glasgow, UK; c University of Ferrara, Cardiology, St Anna Hospital, Ferrara, Italy; d University of Padova, Nephrology, University Hospital, Padova, Italy; e Cardiology, Ospedale Riabilitativo di Alta Specializzazione di Motta di Livenza (TV), Italy; f Cardiology, Ca’Foncello Hospital, Treviso, Italy

**Keywords:** Echocardiography, Congestion, Coronary sinus, Inferior vena cava, Hemodialysis

## Abstract

**Background:**

Congestion predicts a poor prognosis, but its assessment is challenging in clinical practice and requires a multiparametric approach. We investigated if the coronary sinus (CS) diameter can predict mortality in a human model of rapid fluid unloading.

**Methods:**

We measured by echocardiography the CS, and the inferior vena cava (IVC) for comparison, in 60 patients with end-stage chronic kidney disease (ESKD) immediately before and after hemodialysis (HD; age 76 [57–81] years, 40% female, left ventricular ejection fraction 57 [53–56]%). Patients were prospectively followed up for all-cause mortality.

**Results:**

HD-induced decongestion decreased the maximum diameters of both CS and IVC (p ≤ 0.001 for all). The maximum diameter of the CS (CS_max_) was as accurate as the IVC maximum diameter and collapsibility for the identification of congestion, defined as pre-hemodialysis status (AUROC CS_max_ = 0.902 vs IVC = 0.895, p = n.s.). A CS_max_ diameter after hemodialysis > 9 mm predicted all-cause mortality at 12 months (Log-rank Chi square = 11.49, p < 0.001).

**Conclusions:**

A persistently dilated CS after hemodialysis is a marker of residual congestion and predicts death at one year in high-risk ESKD patients.

## 1 Introduction

Venous and tissue congestion is the main cause for hospitalization and predicts a poor outcome in patients with heart failure [1], [2], [3], [4]. However, its clinical assessment is challenging and relies on multiple approaches, including clinical signs and symptoms, measurement of natriuretic peptides, bioimpedance and point-of-care ultrasound-based assessment of the lungs, renal and hepatic venous Doppler, jugular vein distension and, more commonly, inferior vena cava (IVC) diameter and its inspiratory collapse [5], [6], which are accepted surrogates of right atrial pressure (RAP) [7], [8].

In a recent proof-of-concept study in patients on hemodialysis (HD), in whom the assessment of IVC estimates dry weight and predicts congestion-related adverse outcomes [9], [10], [11], [12], [13], changes in IVC diameter were reported to be more sensitive than venous pressure in tracking ultrafiltration volumes [14]. However, IVC measurements can be unreliable or unfeasible in several circumstances, when its diameter and collapsibility are independent of volume status and/or when the subcostal acoustic window is poor [15]. Therefore, as each single method has intrinsic limitations, the assessment of congestion currently still relies on a multiparametric assessment based on clinical, biochemical and ultrasound findings [6].

The coronary sinus (CS) is the largest cardiac vein draining into the right atrium (RA). Owing to its intra-pericardial location, its diameter is not affected by respiration, intrabdominal pressure or splanchnic capacitance. Moreover, it can be easily visualized on echo windows other than the subcostal, thus extending the feasibility of measurement of its diameter [16]. The latter exhibits distinct changes in phase with atrial contraction (Supplemental Fig. 1) and its maximum value (CS_max_) correlates with RAP in patients with right-sided heart disease secondary to pulmonary hypertension [17], [18]. Furthermore, it was found to be dilated in patients with heart failure compared to controls [19], [20]. Nonetheless, it has never been used to track congestion and changes in fluid status, and predict survival in high-risk patients.

We hypothesized that the CS_max_ can predict outcome in a cohort of consecutive patients with end-stage kidney disease (ESKD) undergoing chronic hemodialysis (HD), as a model of rapid intra-patient transition from congested to de-congested state. Specifically, we set out to determine the accuracy of CS_max_ in the identification of congestion and its prognostic value on markedly different volume conditions (i.e., before and after HD), for predicting one-year mortality in this high-risk cohort of patients with ESKD.

## 2 Methods

All study procedures were conducted in accordance with local regulations and practice and with the Declaration of Helsinki. The study protocol was approved by the ethical committee at our institution (ref. 204N/AO/22). Informed consent has been obtained from all participants.

### 2.1 Study participants and HD treatment

Consecutive consenting adult ESKD patients undergoing regular day-time hospital-based HD at the Nephrology Unit of a tertiary hospital were recruited between October 2020 and April 2021. Exclusion criteria were severe valve disease, a history of valve surgery or cardiac transplantation, advanced atrio-ventricular block, persistent left superior vena cava [16], any major congenital heart disease, or lack of capacity. Patients were prospectively followed up for the endpoint of all-cause mortality for a median of 12 months (IQ range 10–14 months) after the index HD session.

Relevant anthropometric, demographic, and clinical characteristics were recorded at the time of the HD session, along with HD-specific HD parameters, including blood pressure (BP) and heart rate before and after treatment.

Total HD fluid extraction for each patient was set by the attending nephrologist (GS, FN) independent of any echocardiographic parameter, based on clinical judgement and on consolidated patient-specific HD protocols, determined clinically and/or by bioimpedance on previous HD session. Relative fluid stability of the HD regimen was tested by comparing fluid extraction on the index HD session with fluid extraction on an independent HD session ≥ 6 months apart.

### 2.2 Echocardiography

Two-dimensional echocardiograms were performed within one hour before and after the HD session using a Philips Epic X5 with a 2.5 MHz transducer by two experienced cardiologists (AB and LR), blind to the biochemical and clinical data of the patients. The echocardiographic examination, including M-mode, 2-D, and Doppler echocardiography, was performed in accordance with the ASE/ESC guidelines [7] (expanded methods are available in the Supplemental Material).

The maximal (expiratory) IVC diameter (IVC_max_) and its percentage decrease in response to sniff inspiration (collapsibility index, IVC_C%_) were assessed as established measures of congestion. IVC diameters were measured in the subcostal window, with the patient lying supine at 1.0 to 2.0 cm from the junction with the right atrium, using the IVC long-axis view and the ultrasound beam perpendicular to the IVC wall [7], [21]. The CS was identified in the atrio-ventricular groove in a 4-chamber posterior apical view (Supplemental Fig. 1). Its diameter was measured from inner-edge to inner-edge within 1 cm from its orifice in the right atrium, with zoom M-mode magnification to obtain a sharper definition of its edges using imaging 2-D echo as guide. Measurements were taken at 2 points in the cardiac cycle: 1) at maximum CS diameter (CS_max_) at the end of ventricular systole and 2) at minimum CS diameter during atrial contraction (CS_min_) (Supplemental Fig. 1). For these measurements, the average value of 5 to 10 consecutive cardiac cycles was derived. A CS collapsibility index (CS_C%_) was calculated as (CS_max_ - CS_min_)·100/CS_max_, for comparison with IVC_C%_.

The reproducibility of IVC measurements is established [22]. In our hands, the intra- and inter-observer variability (variation coefficient) of CS_max_ were < 5 % and 10 %, respectively.

### 2.3 Statistical methods

The statistical analysis was performed with SPSS 28 for Mac (SPSS Italy Inc., Bologna, Italy), Prism (vers. 9.3 for Mac, GraphPad Software, San Diego, California USA, ww.graphpad.com) and MedCalc (vers. 20.023, MedCalc Software Ltd, Ostend, Belgium).

The distribution of categorical variables, expressed as percentage, was compared by chi-square test. Distribution of all continuous variables and deviation from a normal distribution was assessed by graphical plotting and by Kolmogorov-Smirnov test. Data are presented as mean ± SD or median and interquartile range, as appropriate. Skewed data were log transformed or assessed with non-parametric tests, as appropriate. Wilcoxon signed-ranks test was used for paired repeated measurements and univariate correlations were assessed using Spearman’s r throughout, unless otherwise stated.

Multivariate-adjusted comparisons (ANCOVA) and hierarchical linear regression models with backward approach were used to identify determinants of CS_max_, and of corresponding IVC parameters and CS_C%_ for comparison. The models included clinically relevant covariates or statistically significant covariates after appropriate transformation to attain normal distribution, constrained to 10 patients per variable, to avoid overfitting of the models.

Kruskall-Wallis test followed by post-hoc tests, and a parametric test for linear trend (GraphPad Prism) for CS_max_, were used to compare quantitative variables across quartiles of HD-extracted fluid volume.

The area under the Receiver Operator Characteristics (AUROC) curves was used to assess the accuracy of the CS_max_, and of IVC_max_ and IVC_C%_ as the relevant comparators [23], for the identification of fluid overload in the subgroup of patients with > 1.5 L fluid excess before HD [24] (corresponding to the upper three quartiles of HD volume removal). Fluid overload was defined as the pre-dialysis status, as opposed to its resolution after dialysis. The Youden index analysis was used to identify the value that provided the highest accuracy, i.e the best trade-off of sensitivity and specificity.

The impact of CS_max_, and of IVC data for comparison, on cumulative mortality as a function of time, was investigated using Kaplan-Meier curves after dichotomization of data based on Youden-index identified cut-off values, compared by log-rank test, as well as univariate and multivariate Cox regression analysis, with similar statistical constraints as detailed above. Significance was set at p < 0.05 for all tests.

## 3 Results

### 3.1 Study participants and dialysis treatment

From October 2020 to April 2021, we recruited 60 consenting ESKD patients (40 % female; age 76 [57–81] years) undergoing maintenance HD on a thrice-weekly regimen. The HD vintage at time of recruitment was 33 (15–77) months. The demographic and clinical characteristics of the patients are summarized in Supplemental Table 1. Of note, 57 % of the patients had left ventricular hypertrophy; 95 % of patients had a left ventricular ejection fraction ≥ 40 %; 33 %, 27 % and 7 %, were in NYHA functional class II, III and IV, respectively. Persistent atrial fibrillation was found in 13 %.

All participants were on a stable clinically- and bioimpedance-guided unloading protocol from the preceding dialysis sessions prior to recruitment. Stability of the fluid overload across HD sessions was confirmed on study-unrelated visits at ≥ 6 months after recruitment (r_volume extraction at index · follow-up HD session_ = 0.604, p < 0.001). During an HD index session of 3 h on average (range 2–4), the amount of extracted fluid volume was 2500 ml (interquartile range: 2000–3500 ml).

### 3.2 Effects of hemodialysis on hemodynamic and echocardiographic parameters

HD lowered BP (Δ mean BP = -9 ± 16 mmHg, p < 0.001), systolic pulmonary arterial pressure, atrial dimensions, measures of intracardiac pressure and, to a statistically significant but clinically minimal extent, left ventricular cardiac index. At variance, it increased heart rate (Δ HR = 5 ± 8 bpm, p < 0.001), left ventricular ejection fraction and measures of right ventricular function (Table 1).

**Table 1:** Intradialytic hemodynamic and cardiac changes.

	**Baseline**	**After dialysis**	**p**
**Haemodynamics**			
HR, bpm	68 ± 12	74 ± 13	**< 0.001**
SBP, mmHg	139 ± 25	121 ± 23	**< 0.001**
DBP, mmHg	76 ± 14	71 ± 12	**0.039**
MBP, mmHg	97 ± 16	88 ± 14	**< 0.01**
LV Cardiac Index, ml/min/m^2^	1.79 (1.48–2.40)	1.73 (1.45–2.30)	**0.038**
SVRI, dyn·s/cm^5^	1955 (1598–2780)	1965 (1607–2453)	0.730
**Left heart**			
LV EDV, ml/m^2^	47 (42–60)	44 (38–57)	**<0.001**
LV EF (%)	57 (53–62)	60 (56–65)	**<0.001**
LA ESA, cm^2^	21 (16–25)	20 (16–24)	**<0.001**
LA ESV, ml/m^2^	35 (25–40)	32 (25–38)	**<0.001**
E/A ratio, mitral valve (avg)	0.70 (0.56–0.86)	0.70 (0.56–0.84)	0.554
E/E’ ratio, mitral valve (avg)	10 (7–13)	9 (7–12)	**0.006**
**Right Heart**			
RV EDA, cm^2^	19.1 ± 3.8	17.3 ± 3.6	**<0.001**
RV FAC, %	39 ± 7	42 ± 7	**<0.001**
RA ESA, cm^2^	19 (16–22)	18 (15–22)	**<0.001**
RA ESV, ml/m^2^	33 (25–37)	32 (25–38)	**<0.001**
E/A ratio, tricuspidal valve	0.80 (0.70–1.00)	0.80 (0.60–1.00)	0.507
E/E’ ratio, tricuspidal valve	8 (6–10)	6 (5–10)	**0.005**
s’ peak velocity T (cm/s)	11 (9–13)	12 (10–15)	**<0.001**
sPAP (mmHg)	35 (30–45)	25 (25–35)	**<0.001**
TAPSE (mm)	21.6 ± 5.3	23.9 ± 5.6	**<0.001**
TAPSE/sPAP ratio	0.63 (0.38–0.80)	0.90 (0.67–1.08)	**<0.001**
**Inferior Vena Cava**			
IVC_max_, mm	20.0 (17.0–21.0)	13.0 (11.0–17.0)	**<0.001**
IVC_C%_, %	40 (30–50)	60 (50–70)	**<0.001**
**Coronary Sinus**			
**CS_max_, mm**	11.5 (10.0–13.0)	8.0 (7.0–10.0)	**<0.001**

Data are reported as mean ± SD or median (interquartile range). HR = heart rate; SBP, DBP and MBP = systolic, diastolic and mean blood pressure, respectively; LV = left ventricle; SVRI = systemic vascular resistance index; EDV = end diastolic volume, EF = ejection fraction, ESA = end systolic area, ESV = end systolic volume; EDA = end diastolic area; FAC = fractional area change; sPAP = estimated systolic pulmonary arterial pressure; IVC_max_ = inferior vena cava maximum (end-expiratory) diameter; IVC_C%_ = inferior vena cava expiratory-inspiratory collapsibility index; CS_max_ = coronary sinus maximum diameter; CS_min_ = coronary sinus minimum diameter.

### 3.3 Predictors of coronary sinus and impact of hemodialysis

CS maximal diameter (CS_max_) was independently predicted by age and BMI (univariate correlations: Spearman r = 0.271 and r = 0.263, respectively; p < 0.05 for both; see Supplemental Table 2, models A and B for multivariate regression) but not sex, BP, heart rate, hemoglobin, diagnosis of coronary artery disease, or HD vintage. CS_max_ was larger in diabetic patients (p = 0.032) but not after correction for BMI (Supplemental Table 2, models A and B).

**Fig. 1:**
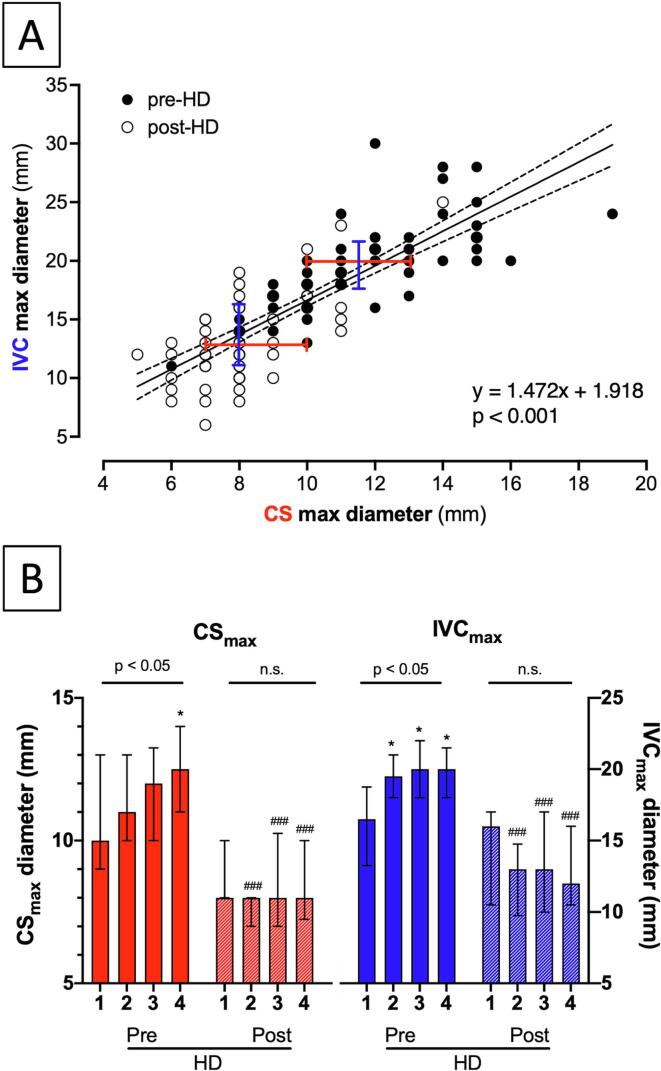
**Maximum diameter of CS and IVC before and after hemodialysis (HD).***Panel A: Correlation plot between pre-dialysis (●) and post-dialysis (○) maximum diameters of CS and IVC* (pre/post-HD CS_max_ and IVC_max_, respectively; Spearman ρ = 0.86 for the aggregate, p < 0.0001). Since the regression slopes do not differ between pre/post HD conditions (p = 0.08) a single regression line with 95 %CI is plotted for all data. Median values and IQR for pre and post-dialysis of CS and IVC are shown in red and blue, respectively. *Panel B: distribution across quartiles of HD-extracted fluid volume.* Data presented as median, interquartile range. Pre-dialytic max diameters of both CS (left, red) and IVC (right, blue) increased across quartiles of fluid volume extraction (1 to 4); the distribution of post-dialytic CS_max_ (dashed columns) was significantly reduced in quartiles 2-to-4 of HD-extracted fluid volume. Likewise, the post-dialytic IVC_max_ was flattened to significantly lower values. * p < 0.05, ** p < 0.01, *** p < 0.001 vs reference pre-dialytic quartile I; ## p < 0.01, ### p < 0.001 vs corresponding pre-dialytic value. (For interpretation of the references to colour in this figure legend, the reader is referred to the web version of this article.)

The additional variable that independently predicted CS_max_ (R^2^ = 0.193, p = 0.002) was the volume of HD extracted fluid, a surrogate for baseline congestion (adjusted B = 0.33). Similar independent associations with extracted volumes were found for inferior vena cava maximum diameter (IVC_max_; R^2^ = 0.216, p < 0.001, along with atrial fibrillation (AF) at the time of the exam) and respiratory collapsibility (IVC_C%_; R^2^ = 0.146, p = 0.003; Supplemental Table 2, models C and D). Both before and after dialysis, CS_max_ and IVC_max_ showed a highly significant correlation with each other (Fig. 1A), and moderate with IVC_C%_ (Supplemental Fig. 2).

Fluid unloading by HD decreased the maximum diameter of both CS and IVC (-26.5 ± 13.2 % and −27.8 ± 19.6 %; Table 1) and increased IVC collapsibility. Changes in CS diameters, IVC_max_ and IVC_C%_ were independent of the AF status (Supplemental Table 3). Before HD, both CS_max_ and IVC_max_ increased with quartiles of fluid volume extraction (for linear trend p = 0.029 for CS_max_ and p = 0.042 for IVC_max_); after HD, they did not differ across quartiles (p = 0.872 and p = 0.862, respectively; Fig. 1B).

The CS diameter showed no variation with respiration, at variance with IVC. Pre-dialysis CS collapsibility (CS_C%_) during the cardiac cycle was predicted by BMI, age, and the volume of HD extracted fluid (Supplemental Table 2, models A and B), similar to CS_max_. However, the strongest predictor of CS_max_ was AF at the time of the exam: the cyclical CS collapsibility was lost before and after HD in the eight patients with permanent AF (Supplemental Table 2). CS_C%_ was unrelated to other CS or IVC measures, except for a tight correlation with post-HD CS_C%_ (Supplemental Fig. 2); after HD, CS_C%_ remained lower in patients in the highest quartiles of fluid volume extraction (Supplemental Fig. 3).

### 3.4 Accuracy of the coronary sinus max diameter for identification of HD volume status

**Fig. 2:**
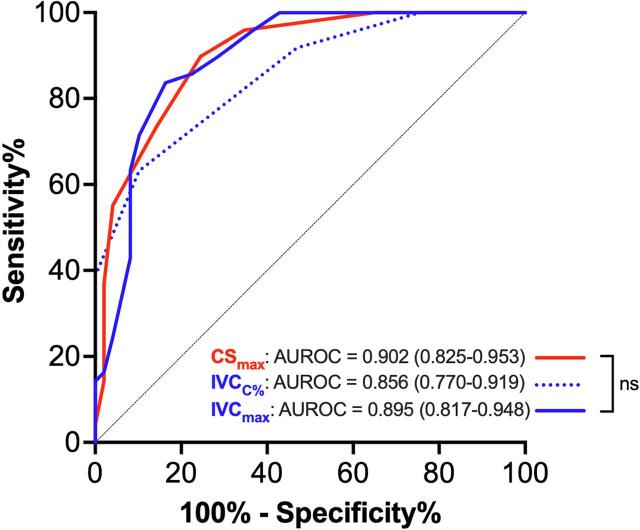
**ROC curve analysis for the accuracy of CS and IVC for the identification of volume excess.** The values of the area under the curve (AUROC), which estimates the overall accuracy, are presented with 95 % confidence intervals. CS_max_ was not inferior to IVC_max_ and IVC_C%_ for identification of congestion (defined as pre-HD state) in patients with HD fluid extraction > 1.5L (n = 49); for all three, p < 0.0001 vs random assessment (i.e. area under the identity line = 0.5); ns = not significant for comparison of AUROC values by Hanley method.

Both IVC_max_ and IVC_C%_ accurately identified a pre-HD state of congestion in the sub-cohort of patients in the upper three quartiles of fluid removal, where the extraction volumes were > 1.5 L (AUROC = 0.895 and AUROC = 0.856, p < 0.0001 vs random for both, Fig. 2B; whole cohort analysis provided as Supplemental Figure 4). CS_max_ was not inferior to IVC parameters to predict fluid overload (AUROC = 0.902, p < 0.0001 vs random assessment; p = ns vs IVC_max_ and IVC_C%_). The CS_max_ value that best discriminated congested from non-congested patients was 9.0 mm, with 89.8 % sensitivity and 75.5 % specificity (Fig. 2). At variance with IVC_C%_, CS_C%_ performed only minimally better than random assessment (AUROC = 0.611, 95 % CI = 0.508–0.708) and worse than CSmax and IVCmax (p < 0.001; not shown).

### 3.5 Prognostic value of congestion assessed by CS

Over a median follow-up of 12 months (IQ range 10–14 months) 10 deaths occurred.

**Fig. 3:**
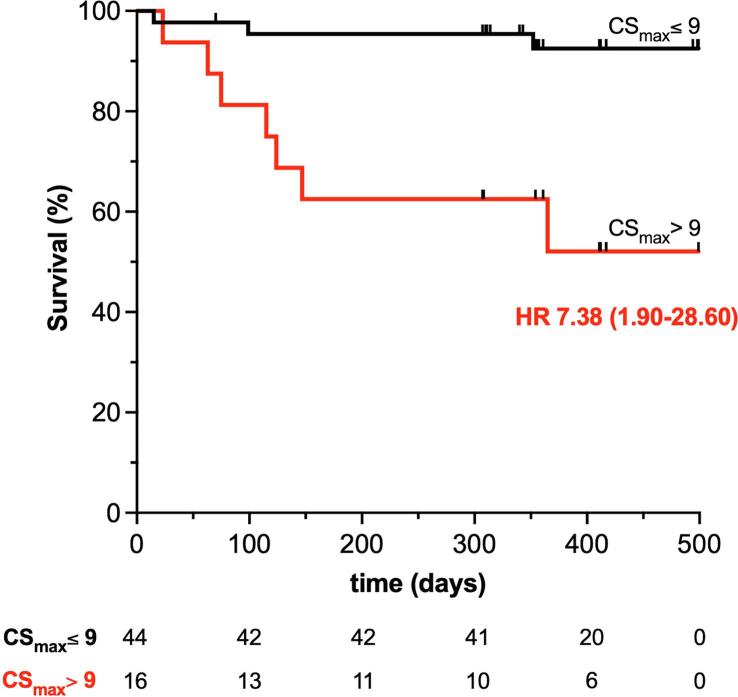
**Probability of survival according to post-dialysis CS maximum diameter.** The Kaplan-Meier curves show that residual congestion, estimated by a post-dialysis CS_max_ larger than 9 mm, i.e. the optimal cut-off identified above for discrimination of congestion vs decongestion, was associated with an excess mortality risk (Log-rank Chi square = 11.49, p < 0.001; n. at risk: 44 for CS_max_ ≤ 9 mm [*black*] and 16 for CS_max_ > 9 mm [*red;* univariate Hazard Ratio (HR) at Cox Regression is presented with 95 % confidence interval, p = 0.004]; two patients were censored in the first group at the time of renal transplantation). (For interpretation of the references to colour in this figure legend, the reader is referred to the web version of this article.)

Larger pre-dialysis and post-dialysis CS_max_ predicted these events at univariate analysis (HR 1.32, 95 %CI: 1.02–1.70, p = 0.033 and HR 1.68, 95 %CI: 1.16–2.43, p = 0.006, respectively); for post-dialysis but not pre-dialysis CS_max_, the prediction was independent of established risk factors in this population, i.e. age, HD vintage, BMI, atrial fibrillation or CAD and left ventricular ejection fraction (HR 1.60, 95 %CI: 1.16–2.19, p = 0.004 and HR 1.35, 95 %CI: 0.99–1.84, p = 0.061, respectively; Supplemental Table 4). When measured after HD, CS_max_ optimal cut-off for the identification of fluid excess (i.e., CS_max_ > 9 mm) identified a subgroup of patients with significantly higher mortality (Fig. 3).

Post-dialysis IVC_max_ offered a similar prediction at univariate (HR 1.19, 95 %CI: 1.03–1.37, p = 0.018) but not multivariate analysis (p = 0.095; Supplemental Table 4). Neither pre-dialysis IVC_max_ (univariate HR 1.08, 95 %CI: 0.92–1.28, p = 0.331) nor IVC_C%_ (or CS_C%_) provided any significant prognostic information.

## 4 Discussion

This study provides compelling evidence that the CS_max_ measured after HD has remarkable prognostic value in predicting all-cause mortality in the extremely high-risk patients with ESKD, independent of other established risk factors like age, HD vintage, BMI, left ventricular ejection fraction, or the presence of atrial fibrillation or CAD [25]. Based on the independent prediction of CS_max_ by a surrogate of congestion (i.e., the volume of fluid extracted with HD) and the accuracy of CS_max_ in its identification, these results are in keeping with the adverse prognostic role of even subtle congestion in patients with heart failure [1], [2], [3], [4] and chronic kidney disease [11], [12], [13], which is increasingly identified with approaches of point-of-care ultrasound [26], [27], [28], [29]. On the whole, our findings suggest that ultrasound measurement of CS_max_ can represent one additional tool for clinicians in the multimodal assessment of congestion. Whether this is incremental in comparison with univariate approaches (e.g., IVC ultrasound measurement, which can be difficult, or even flawed, in patients with cardiac diseases impeding venous return, obesity, lung hyperinflation, and/or during ventilation with increased abdominal pressure or with different breathing patterns [15]) remains to be determined. Nonetheless, the strong prognostic value and the possible cardiac-specific insights offered by the CS_max_ appear promising and worth investigation in other cohorts of high-risk patients.

The suggestion that the measurement of CS diameter is a marker of congestion is supported by previous studies. Patients with congestive heart failure were shown to have larger CS diameters than controls on echo, alongside a reduction of its collapsibility during atrial contraction, by *D’Cruz et al.* [19]. The findings were in keeping with an earlier necroscopy study [30]. In similar patients, Cakici et al linked the CS diameter to measures of right heart dimensions and RV function [20], and in patients with pulmonary hypertension its association with RAP was quantitatively validated by right heart catheterization [17], [18]. However, all these observational studies lacked a longitudinal design. Thus, our study is novel in that it demonstrated: a) dynamic volume-dependent intra-patients changes of the CS diameter, in the unique model of rapid hemodynamic unloading offered by HD in ESKD; b) but also that residual congestion measured by CS ultrasound is a reliable predictor of hard outcomes. We speculate that in the future this could possibly help tailored decongestion strategies.

Importantly, we could measure the maximum CS diameter in all patients both before and after dialysis, i.e. under congested and (presumably) decongested state, thus showing the accessibility of measure and its repeatability for point-of care monitoring purposes. The CS_max_ correlated with IVC diameter and collapsibility before and after dialysis. This was not the case for CS collapsibility: in sinus rhythm patients, CS diameter is known to vary in phase with the cardiac cycle, peaking (CS_max_) during ventricular systole and reaching a nadir during atrial contraction. Unsurprisingly, CS_max_ and its cyclical collapsibility were unaffected by respiration, but influenced by AF: the lack of atrial contraction during the arrhythmia was the main predictor of CS_C%_, as previously reported [19]. Of note, the decrease in CS_max_ after systemic decongestion and the main study conclusions were irrespective of AF.

The lack of simultaneous invasive hemodynamic assessment, which is unethical in the fragile patients investigated, could be seen as a limitation in this study. However, their rapid transition from a fully congested condition (before HD) to a decongested, or less congested, state (after HD) is a reasonable surrogate for invasive assessment of volume status and IVC diameter, which is held to be a sensitive non-invasive tool validated in the ESKD population [14] and has been used for comparison throughout the present analysis. Moreover, a cross-sectional validation of CS measurement against catheter-based RAP has already been provided [18]. Whether this association with RAP underscores information different from IVC dimensions, as the CS intra-pericardial location and recent findings on RAP-independent IVC changes would suggest [31], remains to be proven. The relatively small sample size of our single-center study might also be regarded as a limitation and could explain why some known clinical predictors of death [25], [32] did not achieve statistical significance, at variance with the CS diameter. Caution is advised in generalizing these proof-of-principle findings from our high-risk cohort, and additional longitudinal studies in heart failure patients are warranted. Finally, while the impact of AF on CS collapsibility was conclusively confirmed by our data, only 13 % of our patients had AF at the time of the exam and these future studies should include a more representative proportion of such patients.

In conclusion, this study showed that CS maximum diameter measured by ultrasound identified congestion, and predicted all-cause mortality at 12 months when assessed after HD, suggesting insufficient decongestion in this high-risk cohort. Based on these important findings, a prospective randomized clinical trial aimed at comparing death-free survival between post-HD CS_max_-guided and traditional non CS_max_-guided decongestion has been started (ref. 5802/AO/23).

Funding

The authors are funded by the Ministero dell’Università e della Ricerca (MIUR; Dotazione Ordinaria della Ricerca dipartimentale) and by FORICA (The FOundation for advanced Research In Hypertension and CArdiovascular diseases).

## Declaration of Competing Interest

The authors declare that they have no known competing financial interests or personal relationships that could have appeared to influence the work reported in this paper.
