# Supplementary material for: Coronary sinus diameter to estimate congestion and predict survival
Source: Int J Cardiol Heart Vasc. 2023 Nov 8;49:101294. doi: 10.1016/j.ijcha.2023.101294 (PMC10663896; doi:10.1016/j.ijcha.2023.101294)
Supplement: Supplementary data 1 [file mmc1.docx]

**Coronary Sinus Diameter to Predict Survival**

SUPPLEMENTAL MATERIAL

**INDEX**

**Page 2. Expanded Methods**

**Page 4 Supplemental Figure 1.** Coronary sinus identification and measure

**Page 5 Supplemental Figure 2.** Correlations between CS and IVC

**Page 6 Supplemental Figure 3.** Distribution of CS collapsibility and maximum diameter before and after HD, across quartiles of HD-extracted fluid volume, for patients on sinus rhythm only (n=52)

**Page 7 Supplemental Figure 4.** ROC curve analysis for the accuracy of IVC and CS in the identification of volume status in the entire study cohort (n=60).

**Page 8 Supplemental Table 1.** Patient Characteristics

**Page 9 Supplemental Table 2.** Coronary sinus (CS) and inferior vena cava (IVC) covariates

**Page 11** **Supplemental Table 3.** Intradialytic SC and IVC changes according to cardiac rhythm at echo

**Page 12 Supplemental Table 4**. Multivariable Cox regression analysis for all-cause mortality

**Page 13 Supplemental References**

**Expanded Methods**

**Echocardiography protocol and hemodynamics**

Two-dimensional echocardiograms were performed using a Philips Epic X5 with a 2.5 MHz transducer by the same two highly experienced cardiologists (AB and LR), blind to the biochemical and clinical data of the patients. The echocardiographic examination, including M-mode, 2-D, and Doppler echocardiography, was performed in accordance with the ASE/ESC guidelines.^1^

The left ventricle (LV) ejection fraction (EF) was estimated from the apical 4- and 2-chamber views using the Simpson’s biplane method. The LV wall thickness and the left atrial dimension were measured from the parasternal long-axis view. The LV mass index was estimated by Devereux’s formula with normalization by height (in meters) to the 2.7 power to avoid underestimation in overweight-obese patients^2^. Left ventricular hypertrophy was defined as LV mass index >50 g/m^2.7^ and >47 g/m^2.7^ for men and women, respectively.^3^

The maximal (expiratory) IVC diameter (IVC_max_) and its percentage decrease in response to sniff inspiration (collapsibility index, IVC_C%_) were assessed as established measures of congestion; IVC diameters were measured in the subcostal window, with the patient lying supine at 1.0 to 2.0 cm from the junction with the right atrium, using the IVC long-axis view and the ultrasound beam perpendicular to the IVC wall. The CS was identified in the atrio-ventricular groove in a 4-chamber posterior apical view (Supplemental Figure 1). Its diameter was measured from inner-edge to inner-edge within 1 cm from its orifice in the right atrium, with zoom M-mode magnification to obtain a sharper definition of its edges using imaging 2-D echo as guide. Measurements were taken at 2 points in the cardiac cycle: 1) at maximum CS diameter (CS_max_) at the end of ventricular systole and 2) at minimum CS diameter during atrial contraction (CS_min_) (Supplemental Figure 1). For these measurements, the average value of 5 to 10 consecutive cardiac cycles was derived. CS collapsibility index (CS_C%_) was calculated as (CS_max_ - CS_min_)·100/CS_max_.

Mean arterial pressure was calculated as diastolic blood pressure + 1/3 of pulse pressure. Cardiac output was calculated as product of stroke volume and heart rate; cardiac index as body surface area-adjusted cardiac output.

A systemic vascular resistance index was calculated as product of mean arterial pressure and 80/cardiac index; arterial compliance was estimated by the stroke volume/pulse pressure ratio; stroke work as product of systolic blood pressure (pressure load) and stroke volume (volume load) and converted into gram-meters per beat by multiplying for the conversion factor 0.0014.^4^

**Supplemental Figure 1**

**Coronary sinus identification and measure.** 2D echo- and ECG-guided zoom M-mode image of the coronary sinus diameter and its collapsibility during the cardiac cycle. The upper 2D panel depicts a 4-chamber posterior apical view: LV = left ventricle; RV = right ventricle; RA = right atrium; white arrowhead = orifice of the coronary sinus entry into the RA.

**Supplemental Figure 2**

**Correlations between CS and IVC measures.** *Left panel:* Heatmap showing Spearman ρ (red-to-blue scale) and significance of correlation (grey scale) between maximal diameter and collapsibility index of coronary sinus (CS) and inferior vena cava (IVC), before and after dialysis; for significant correlations (p<0.05), the value of Spearman ρ is superimposed.

**Supplemental Figure 3**


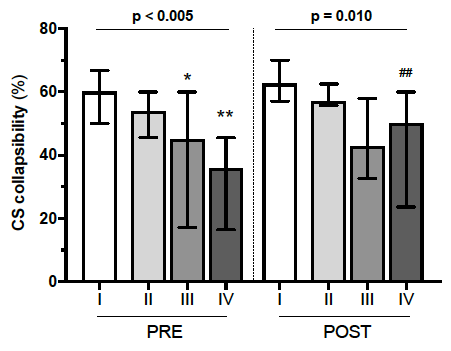


**Distribution of CS collapsibility before and after HD, across quartiles of HD-extracted fluid volume.** Data presented as median, interquartile range. Pre-dialytic CS_C%_ was progressively lower with increasing quartiles (I to IV) of fluid volume extraction; such distribution persisted for post-dialytic values, despite an overall increase.. * p < 0.05, ** p < 0.01, *** p<0.001 vs reference pre-dialytic quartile I; ## p < 0.01, ### p<0.001 vs corresponding pre-dialytic value.

**Supplemental figure 4**

**ROC curve analysis for the accuracy of IVC and CS in the identification of volume status** **in the entire study cohort (n=60), regardless of extraction volumes.** The values of the area under the curve (AUC), which estimates the overall accuracy, are presented with 95% confidence interval. CS_max_ was not inferior to IVC_max_ and IVC_C%_ for the identification of congestion (defined as pre-HD state); for all three p<0.0001 vs random assessment (i.e. the area under the identity line, AUC = 0.5). The CS_max_ value that offered the best discrimination between congested and non-congested conditions (Youden Index) was 8.0 mm, with 95.0% sensitivity and 63.3% specificity. CS_%_ performed only modestly better than random assessment and significantly worse than the others.

**Supplemental Table 1. Patient characteristics**

| Age, yr | 76 (57-81) |
| --- | --- |
| Men, n (%) | 36 (60) |
| Height, cm | 170 (162-175) |
| BMI, Kg/m^2^ | 24.7 (22.5-29.1) |
| BSA, m^2^ | 1.83 ± 0.22 |
| Hypertension, n(%) | 55 (92) |
| Polycystic kidney disease, n(%) | 8 (13) |
| Diabetes Mellitus, n (%) | 16 (27) |
| Coronary Artery disease, n(%) | 23 (38) |
| Atrial Fibrillation, n(%) | 8 (13) |
| NYHA class I/II/III/IV, n(%) | 20/20/16/4 (33/33/27/7) |
| Left ventricular ejection fraction (EF), biplane (%)  Preserved EF (≥50%); n (%)  Mildly reduced EF (≥40 and < 50%); n (%)  Reduced EF (<40%); n (%) | 57 (53-62)  50 (83)  7 (12)  3 (5) |
| Hemoglobin, g/L | 110 ± 9 |

Data presented as n (%), mean ± SD or median and interquartile range, as appropriate.

**Supplemental table 2. Coronary sinus (CS) and inferior vena cava (IVC) covariates**

| Dependent Variable: **predialysis CS max diameter (mm)** | | | | |  |  |  | |
| --- | --- | --- | --- | --- | --- | --- | --- | --- |
|  | | | |  | standardized | **Sig.** | 95% CI for B | |
| **Model** |  |  | **B** | SE | Beta |  | Lower limit | Upper limit |
| **A** | R^2^ = 0.221 | constant | -1,929 | 5,14 |  | 0,709 | -12,235 | 8,377 |
|  |  | **AGE** | **0,061** | 0,023 | 0,361 | **0,010** | 0,016 | 0,107 |
|  |  | HD vintage | **-0,001** | 0,005 | -0,032 | 0,797 | -0,011 | 0,008 |
|  |  | SEX (1M,2F) | **-0,991** | 0,591 | -0,212 | 0,099 | -2,176 | 0,194 |
|  |  | AF (0N,1Y) | **0,668** | 0,884 | 0,099 | 0,453 | -1,105 | 2,441 |
|  |  | CAD (0N,1Y) | **-0,332** | 0,588 | -0,07 | 0,575 | -1,51 | 0,847 |
|  |  | **BMI (lg10)** | **7,622** | 3,356 | 0,287 | **0,027** | 0,892 | 14,353 |
| **B** | R^2^ = 0.161 | constant | -2,847 | 5,039 |  | 0,574 | -12,937 | 7,243 |
|  |  | **AGE** | **0,056** | 0,021 | 0,332 | **0,009** | 0,015 | 0,098 |
|  |  | **BMI (lg10)** | **7,513** | 3,261 | 0,283 | **0,025** | 0,983 | 14,042 |
| **C** | R^2^ = 0.261 | constant | 3,233 | 5,939 |  | 0,589 | -8,686 | 15,151 |
|  |  | **AGE** | **0,056** | 0,023 | 0,33 | **0,017** | 0,011 | 0,102 |
|  |  | HD vintage | **-0,003** | 0,005 | -0,07 | 0,572 | -0,012 | 0,007 |
|  |  | SEX (1M,2F) | **-0,834** | 0,588 | -0,178 | 0,162 | -2,015 | 0,346 |
|  |  | AF (0N,1Y) | **0,735** | 0,87 | 0,109 | 0,402 | -1,011 | 2,48 |
|  |  | CAD (0N,1Y) | **-0,431** | 0,581 | -0,091 | 0,462 | -1,596 | 0,735 |
|  |  | BMI (lg10) | **3,282** | 4,193 | 0,124 | 0,437 | -5,133 | 11,696 |
|  | ***** | Extracted volume (L) | **0,491** | 0,293 | 0,26 | 0,099 | -0,096 | 1,078 |
| **D** | R^2^ = 0.193 | constant | 6,596 | 1,572 |  | 0,000 | 3,448 | 9,745 |
|  |  | **AGE** | **0,049** | 0,02 | 0,289 | **0,018** | 0,009 | 0,09 |
|  | ***** | **Extracted volume (L)** | **0,627** | 0,224 | 0,332 | **0,007** | 0,178 | 1,077 |

| Dependent Variable: **predialysis IVC max diameter (mm)** | | | | |  |  |  | |
| --- | --- | --- | --- | --- | --- | --- | --- | --- |
|  | | | |  | standardized | **Sig.** | 95% CI for B | |
| **Model** |  |  | **B** | SE | Beta |  | Lower limit | Upper limit |
| **C** | R^2^ = 0.248 | constant | 23,002 | 9,678 |  | 0,021 | 3,582 | 42,421 |
|  |  | AGE | 0,011 | 0,037 | 0,04 | 0,766 | -0,063 | 0,085 |
|  |  | HD vintage | -0,001 | 0,008 | -0,02 | 0,874 | -0,017 | 0,014 |
|  |  | sex (1M,2F) | -0,978 | 0,959 | -0,13 | 0,312 | -2,902 | 0,945 |
|  |  | AF (0N,1Y) | 2,739 | 1,418 | 0,252 | 0,059 | -0,105 | 5,584 |
|  |  | CAD (0N,1Y) | 0,411 | 0,947 | 0,054 | 0,666 | -1,489 | 2,31 |
|  |  | BMI (lg10) | -4,751 | 6,833 | -0,111 | 0,490 | -18,461 | 8,96 |
|  | ***** | **Extracted volume (L)** | **1,31** | 0,477 | 0,43 | **0,008** | 0,353 | 2,266 |
| **D** | R^2^ = 0.216 | constant | 16,226 | 1,034 |  | 0,000 | 14,157 | 18,296 |
|  |  | **AF (0N,1Y)** | **2,715** | 1,277 | 0,25 | **0,038** | 0,157 | 5,273 |
|  | ***** | **Extracted volume (L)** | **1,158** | 0,358 | 0,38 | **0,002** | 0,441 | 1,874 |

| Dependent Variable: **predialysis IVC collapsibility (%)** | | | | |  |  |  | |
| --- | --- | --- | --- | --- | --- | --- | --- | --- |
|  | | | |  | standardized | **Sig.** | 95% CI for B | |
| **Model** |  |  | **B** | SE | Beta |  | Lower limit | Upper limit |
| **C** | R^2^ = 0.191 | constant | 44,612 | 38,73 |  | 0,255 | -33,105 | 122,328 |
|  |  | AGE | -0,128 | 0,148 | -0,121 | 0,390 | -0,425 | 0,169 |
|  |  | HD vintage | -0,016 | 0,031 | -0,067 | 0,605 | -0,079 | 0,046 |
|  |  | sex (1M,2F) | 0,031 | 3,836 | 0,001 | 0,994 | -7,667 | 7,729 |
|  |  | AF (0N,1Y) | -2,87 | 5,673 | -0,068 | 0,615 | -14,253 | 8,514 |
|  |  | CAD (0N,1Y) | -2,815 | 3,788 | -0,096 | 0,461 | -10,416 | 4,787 |
|  |  | BMI (lg10) | 15,695 | 27,344 | 0,095 | 0,568 | -39,175 | 70,566 |
|  | ***** | **Extracted volume (L)** | **-4,879** | 1,908 | -0,415 | **0,014** | -8,708 | -1,05 |
| **D** | R^2^ = 0.146 | constant | 54,276 | 4,097 |  | 0,000 | 46,076 | 62,477 |
|  | ***** | **Extracted volume (L)** | **-4,494** | 1,427 | -0,382 | **0,003** | -7,351 | -1,638 |

| Dependent Variable **predialysis CS collapsibility (%)** | | | | |  |  |  | |
| --- | --- | --- | --- | --- | --- | --- | --- | --- |
|  | | | |  | standardized | **Sig.** | 95% CI for B | |
| **Model** |  |  | **B** | SE | Beta |  | Lower limit | Upper limit |
| **A** | R^2^ = 0.544 | constant | 138,565 | 35,137 |  | 0,000 | 68,088 | 209,041 |
|  |  | **AGE** | **-0,36** | 0,154 | -0,239 | **0,024** | -0,67 | -0,05 |
|  |  | HD vintage | -0,018 | 0,032 | -0,054 | 0,571 | -0,083 | 0,046 |
|  |  | sex (1M,2F) | 3,143 | 3,997 | 0,076 | 0,435 | -4,873 | 11,16 |
|  |  | **AF (0N,1Y)** | **-35,56** | 5,98 | -0,597 | **0,000** | -47,554 | -23,566 |
|  |  | CAD (0N,1Y) | 1,785 | 3,977 | 0,043 | 0,655 | -6,191 | 9,761 |
|  |  | **BMI (lg10)** | **-48,77** | 22,704 | -0,208 | **0,036** | -94,309 | -3,23 |
| **B** | R^2^ = 0.534 | constant | 135,662 | 34,329 |  | 0,000 | 66,892 | 204,432 |
|  |  | **AGE** | **-0,33** | 0,145 | -0,22 | **0,026** | -0,621 | -0,04 |
|  |  | **AF (0N,1Y)** | **-35,93** | 5,709 | -0,603 | **0,000** | -47,366 | -24,495 |
|  |  | **BMI (lg10)** | **-45,277** | 22,012 | -0,193 | **0,044** | -89,372 | -1,181 |
| **C** | R^2^ = 0.587 | constant | 91,958 | 39,269 |  | 0,023 | 13,16 | 170,756 |
|  |  | **AGE** | **-0,312** | 0,15 | -0,207 | **0,042** | -0,613 | -0,011 |
|  |  | HD vintage | -0,005 | 0,032 | -0,014 | 0,879 | -0,068 | 0,059 |
|  |  | sex (1M,2F) | 1,71 | 3,89 | 0,041 | 0,662 | -6,096 | 9,515 |
|  |  | **AF (0N,1Y)** | **-36,171** | 5,752 | -0,607 | **0,000** | -47,713 | -24,629 |
|  |  | CAD (0N,1Y) | 2,692 | 3,841 | 0,065 | 0,487 | -5,016 | 10,399 |
|  |  | BMI (lg10) | -9,004 | 27,725 | -0,038 | 0,747 | -64,638 | 46,631 |
|  | * | **Extracted volume (L)** | **-4,497** | 1,935 | -0,27 | **0,024** | -8,379 | -0,615 |
| **D** | R^2^ = 0.581 | constant | 81,014 | 10,286 |  | 0,000 | 60,41 | 101,619 |
|  |  | **AGE** | **-0,279** | 0,135 | -0,185 | **0,043** | -0,549 | -0,009 |
|  |  | **AF (0N,1Y)** | **-37,103** | 5,335 | -0,623 | **0,000** | -47,792 | -26,415 |
|  | * | **Extracted volume (L)** | **-4,786** | 1,445 | -0,287 | **0,002** | -7,68 | -1,892 |

HD = hemodialysis; AF = atrial fibrillation; CAD = coronary artery disease; BMI = body mass index

**Supplemental Table 3. Intradialytic SC and IVC changes according to cardiac rhythm at echo**

|  | **Baseline** | **After dialysis** | **p** | **∆** (%) |
| --- | --- | --- | --- | --- |
| **Sinus Rhythm, n=52** | | | | |
| **CS_max_, mm** | **11.0 (10.0-13.0)** | **8.0 (7.0-9.0)** | **<0.001** | -27 ± 14 |
| CS_min_, mm | 6.0 (4.0-7.0) | 3.0 (3.0-4.0) | **<0.001** |  |
| CS_C%_, % | 50.0 (40-60.0) | 57 (50-63) | **0.002** |  |
| IVC_max_, mm | 19.5 (17.0-21.0) | 13.0 (11.0-16.5) | **<0.001** | -28 ± 20 |
| IVC_C%_, % | 40 (30-50) | 60 (50-70) | **<0.001** |  |
| **Atrial fibrillation, n = 8** | | | | |
| CS_max_, mm | 12.5 (12.0-14.5) | 10.5 (8.0-11.0)* | **0.011** | -23 ± 11 |
| CS_min_, mm | 11.5 (11.0-13.5)*** | 9.0 (7.5-10.0)*** | **0.011** |  |
| CS_C%_, % | 8 (7-8)*** | 10 (8-14)*** | 0.625 |  |
| IVC_max_, mm | 21.5 (20.0-24.5)* | 15.5 (13.5-21.5)* | **0.017** | -24 ± 16 |
| IVC_C%_, % | 38 (30-50) | 50 (40-65) | **0.027** |  |

Data are reported as median (interquartile range). CS_max_ = coronary sinus maximum diameter; CS_min_ = coronary sinus minimum diameter; CS_C%_ = coronary sinus collapsibility index; IVC_max_ = inferior vena cava maximum (end-expiratory) diameter; IVC_C%_ = inferior vena cava expiraory-inspiratory collapsibility index. * p < 0.05, ** p < 0.01, *** p<0.001 vs corresponding values in patients on sinus rhythm.

**Supplemental Table 4. Multivariable Cox regression analysis for all-cause mortality**

|  |  | **Sig.** | **HR** | **95% CI for HR** | |
| --- | --- | --- | --- | --- | --- |
| **UNIVARIABLE** | AGE | 0.139 | 1.042 | 0.987 | 1.101 |
|  | HD vintage | 0.791 | 0.999 | 0.988 | 1.009 |
|  | AF (0N,1Y) | 0.146 | 2.733 | 0.705 | 10.588 |
|  | CAD (0N,1Y) | 0.792 | 1.186 | 0.333 | 4.217 |
|  | BMI (lg10) | 0.389 | 0.031 | 0 | 81.967 |
|  | LVEF (%) | 0.766 | 0.990 | 0.929 | 1.056 |
|  | SC max > 9 pre-HD | 0.405 | 26.038 | 0.012 | 55679.126 |
|  | **SC max > 9 post-HD** | **0.004** | **7.377** | **1.903** | **28.600** |
|  |  |  |  |  |  |
| **MULTIVARIABLE** | **AGE** | 0.560 | 1.02 | 0.954 | 1.091 |
|  | **HD vintage** | 0.961 | 1 | 0.989 | 1.011 |
|  | **AF (0N,1Y)** | 0.245 | 2.505 | 0.532 | 11.798 |
|  | **CAD (0N,1Y)** | 0.590 | 1.441 | 0.382 | 5.432 |
|  | **BMI (lg10)** | 0.203 | 0.002 | 0 | 28.624 |
|  | **LVEF (%)** | 0.798 | 1.009 | 0.941 | 1.082 |
|  | **SC max pre-HD** | 0.061 | 1.347 | 0.986 | 1.841 |
|  |  |  |  |  |  |
| **MULTIVARIABLE** | **AGE** | 0.442 | 1.029 | 0.956 | 1.108 |
|  | **HD vintage** | 0.935 | 1 | 0.989 | 1.011 |
|  | **AF (0N,1Y)** | 0.856 | 1.166 | 0.222 | 6.117 |
|  | **CAD (0N,1Y)** | 0.666 | 1.368 | 0.33 | 5.663 |
|  | **BMI (lg10)** | 0.524 | 0.039 | 0 | 826.308 |
|  | **LVEF (%)** | 0.606 | 1.021 | 0.944 | 1.103 |
|  | **SC max post-HD** | **0.004** | **1.597** | **1.164** | **2.192** |
|  |  |  |  |  |  |
| **MULTIVARIABLE** | **AGE** | 0.304 | 1.033 | 0.971 | 1.1 |
|  | **HD vintage** | 0.787 | 0.998 | 0.987 | 1.01 |
|  | **AF (0N,1Y)** | 0.29 | 2.566 | 0.448 | 14.684 |
|  | **CAD (0N,1Y)** | 0.676 | 1.342 | 0.337 | 5.344 |
|  | **BMI (lg10)** | 0.286 | 0.004 | 0 | 94.457 |
|  | **LVEF (%)** | 0.761 | 0.99 | 0.926 | 1.058 |
|  | **IVC max pre-HD** | 0.739 | 1.034 | 0.851 | 1.256 |
|  |  |  |  |  |  |
| **MULTIVARIABLE** | **AGE** | 0.385 | 1.029 | 0.965 | 1.098 |
|  | **HD vintage** | 0.952 | 1 | 0.989 | 1.012 |
|  | **AF (0N,1Y)** | 0.484 | 1.857 | 0.328 | 10.512 |
|  | **CAD (0N,1Y)** | 0.516 | 1.554 | 0.411 | 5.872 |
|  | **BMI (lg10)** | 0.445 | 0.017 | 0 | 602.609 |
|  | **LVEF (%)** | 0.899 | 1.004 | 0.938 | 1.076 |
|  | **IVC max post-HD** | 0.095 | 1.159 | 0.975 | 1.378 |

HD = hemodialysis; AF = atrial fibrillation; CAD = coronary artery disease; BMI = body mass index; LVEF = left ventricular ejection fraction; CS = coronary sinus; IVC = inferior vena cava.

**SUPPLEMENTAL REFERENCES**

1. Lang RM, Badano LP, Mor-Avi V, Afilalo J, Armstrong A, Ernande L, et al. Recommendations for cardiac chamber quantification by echocardiography in adults: an update from the American Society of Echocardiography and the European Association of Cardiovascular Imaging. J Am Soc Echocardiogr. 2015 Jan; 28(1):1-39.e14.
2. Devereux RB, Alonso DR, Lutas EM, et al. Echocardiographic assessment of left ventricular hypertrophy: Comparison to necropsy findings. Am J Cardiol. 1986;57(6):450-458.
3. Williams B, Mancia G, Spiering W, et al. 2018 ESC/ESH Guidelines for the management of arterial hypertension. J Hypertens. 2018;36(10):1953-2041.
4. De Simone G, Devereux RB, Kimball TR, et al. Interaction between body size and cardiac workload. Hypertension. 1998;31(5):1077-1082.
